# Editorial: Functional nanogels and multicomponent supramolecular systems

**DOI:** 10.3389/fchem.2024.1526301

**Published:** 2024-11-22

**Authors:** Bruno Pedras, Eduardo R. Triboni

**Affiliations:** ^1^ iBB-Institute for Bioengineering and Biosciences, Instituto Superior Técnico and Associate Laboratory i4HB – Institute for Health and Bioeconomy at Instituto Superior Técnico, Universidade de Lisboa, Lisboa, Portugal; ^2^ Laboratório Nanotecnologia e Engenharia de Processos-NEP, Universidade de São Paulo, Lorena, São Paulo, Brazil

**Keywords:** supramolecular, gel, nanotechnology, multifunctional materials, devices

Supramolecular chemistry is at the forefront of bringing unprecedented precision to functional design, by allowing for the self-assembly of intricate molecular architectures. Contemporary supramolecular chemistry has moved toward conceptions such as molecular information encoding and intermolecular interaction frameworks, resulting in highly complex, “smart” materials with arising properties that naturally manifest on the multi-nanometer scale. In this sense, nanochemistry complements supramolecular chemistry, by providing well-organized combinations at the nanoscale that exhibit distinct advantages compared to larger-scale materials. Both fields have driven advancements in sol-gel chemistry, enabling new synthetic pathways, molecular structures and unique properties.

This multidisciplinary Research Topic aims to present various perspectives on developing multifunctional materials, molecular assemblies and organic-inorganic hybrids. By presenting current trends in supramolecular chemistry and nanochemistry, we intend to highlight contributions across diverse disciplines, showcasing conceptual paradigms, enhanced properties, and practical applications. This set of scientific articles brings together topics such as metal-organic frameworks, coordination cages, cavitands, aerogels and molecular materials that define their properties as a function of intermolecular arrangements or stimulated molecular rearrangements. Six articles are presented: one Mini Review, three Reviews and two Original Research papers, all of them focusing on topics that attract attention when it comes to the use of molecular systems and their scientific and technological impact under the line of historical scope, current demands and perspectives.

Loan et al. reported a novel class of photochromic molecules that exhibit photo-switching in the solid state. The authors synthesized a small library of phenylindole alkene dimers (PIDs) in order to tune their optical properties. These molecules show a rapid conversion between colourless and coloured states, displaying both photo- and thermal reversibility in the solid state, which enabled their application as photo-switchable inks. Other possible applications of these materials comprise writable filters or gratings and, in a biological environment, modulation of drug-receptor interactions.

Gayathry et al. investigated the supramolecular assembly of coumarin 7 with a cyclodextrin for biomolecular applications. The authors noticed that the non-covalent host-guest interaction of the macrocyclic host with both prototropic forms of the coumarin dye produced a considerable enhancement in the fluorescence yield and lifetime of the dye, indicating a confinement of both forms (C7/C7H^+^) in the extended macrocyclic cavity. The utility of the formed complex for bioimaging applications was shown by using confocal imaging of the *Drosophila* fly gut staining.

Liang et al. offer a Mini Review on distinct selectivity inside self-assembled coordination cages. Two representative examples of self-assembled coordination cages are discussed, in which the organic reactions show distinct selectivity when compared to the one found on the outside bulk solution conditions: self-assembled Pd(II)-ligand octahedral and Ga(III)-ligand tetrahedral coordination cages. The cage host forms spontaneously through the coordination of metal ions and appropriate ligands, without requiring covalent bond formation. Despite being water-soluble, its interior is highly hydrophobic, preventing water from entering its cavity. This can significantly modify the mechanism of water-associated processes and originates distinct selectivity in such cases. These examples highlight the remarkable capacity of self-assembled coordination cages to control reaction selectivity.

In the review of Marcos et al., the recent applications of fluorescent homooxacalixarenes in supramolecular systems have been described. The authors review recent advancements (from 2006 onwards) in fluorescent homooxacalixarene-based supramolecular systems, with a special focus on fluorescence sensing using either their intrinsic fluorescence or the extrinsic fluorescence of organic fluorophores—covalently bound to the calixarenes or forming supramolecular complexes with them. This review also offers a discussion of sensing applications of ions, ion pairs and neutral molecules, along with the potential for temperature measurement via thermally activated delayed fluorescence.

In their review on the effect of synthesis conditions and process parameters on aerogel properties, Konuk et al. unveil literature reports investigating the effective parameters from the precursor dissolution step to the supercritical drying step, including the carbonization process for carbon aerogels. The authors expected that their review will provide new insights on understanding aerogel production and changes in the conditions and parameters that impact their properties during each production step, in order to tailor these properties. They highlight that the molecular structure and concentration of precursor and solvent affect not only the gelation time but also the mechanical strength, volumetric shrinkage, bulk density, and size of the pores. Catalyst type, concentration, and pH effects were also discussed.

Cué-Sampedro et al. present a review on supramolecular systems and their connection with metal-organic structures. A general introduction to metal-organic frameworks (MOFs) and supramolecularity is presented, along with a review of key advancements in confined space chemistry, and the relationships between MOFs and supramolecular structures, in order to gain deeper understanding of structure–property relationships.

We are of the strong opinion that the contents of this Research Topic offer readers an overview of the recent progress of these constantly expanding fields, and we expect it to be of great interest to a broad audience.

